# Predictors of EQ-5D-3L outcomes amongst injured Māori: 1-year post-injury findings from a New Zealand cohort study

**DOI:** 10.1007/s11136-022-03085-3

**Published:** 2022-01-25

**Authors:** Brett Maclennan, Emma Wyeth, Ari Samaranayaka, Sarah Derrett

**Affiliations:** 1 Department of Preventive and Social Medicine, Dunedin School of Medicine, Te Roopū Rakahau Hauora Māori a Kāi Tahu (Ngāi Tahu Māori Health Research Unit), University of Otago, PO Box 56, Dunedin, 9054 New Zealand; 2 Biostatistics Centre, Division of Health Sciences, University of Otago, Dunedin, New Zealand

**Keywords:** Māori, Indigenous, Post-injury outcomes, Predictors, Health-related quality of life, EQ-5D-3L

## Abstract

**Purpose:**

Māori, the Indigenous population of New Zealand (NZ), are at higher risk of problems with health-related quality of life (HRQoL) 12 months following injury. This paper examines pre-injury sociodemographic and health characteristics and injury-related factors, including healthcare access, and their association with HRQoL outcomes 12 months after injury.

**Methods:**

The Prospective Outcomes of Injury Study recruited 2856 injured New Zealanders aged 18–64 years from the entitlement claims register of the country’s no-fault injury insurance agency. One-fifth (*n* = 566) of the cohort were Māori. Information on predictors and outcomes, with the exception of injury and hospitalisation, was obtained directly from participants at approximately 3 and 12 months post-injury. The outcomes of interest were responses to the five dimensions of the EQ-5D-3L and a dichotomous measure obtained by summing scored responses to each question. Modified Poisson regression was used to identify predictors of each outcome at 12 months post-injury.

**Results:**

Predictors differed by outcome. Being female, experiencing EQ-5D-3L problems pre-injury, having ≥ 2 chronic conditions pre-injury, perceiving one’s injury to be a threat of long-term disability, and having trouble accessing health services for injury were common predictors of EQ-5D-3L problems at 12 months post-injury for Māori.

**Conclusion:**

Opportunities exist to improve HRQoL outcomes by identifying individuals in the early stages of injury recovery who may benefit from further treatment and support.

## Introduction

Indigenous populations around the world experience significant health inequities compared to non-indigenous populations [1]. Māori, the Indigenous population of New Zealand (NZ), are no exception, experiencing widespread inequities in access to health services, quality of treatment, and health and well-being outcomes [2]. Highlighted and exacerbated by the COVID-19 pandemic [3–5], inequities in health have long been recognised as unfair and unacceptable [6] and elimination of these should be a priority for communities, health providers, and governments [5].

The collection and analysis of high-quality Indigenous health data are integral to achieving health equity. The Prospective Outcome of Injury Study (POIS)—a national longitudinal study of injured New Zealanders—was specifically designed to permit dedicated analyses of Māori data [7]. The POIS ensured recruitment of sufficient numbers of Māori so that robust statistical analyses could be undertaken and that findings would be meaningful, relevant, and of high utility to Māori [8]. Previous analyses of POIS Māori data include estimating the prevalence and, in some cases predictors, of life satisfaction [9], work participation [10], and various other outcomes [11–13] at three-, 12-, and 24-months post-injury.

One outcome measure we have previously reported on for POIS, at three- and 12-months post-injury, is health-related quality of life (HRQoL), measured using the EQ-5D-3L [11, 12, 14]. The EQ-5D-3L is a generic questionnaire developed by the EuroQol Group [15, 16] that includes questions asking respondents to report the extent of problems experienced across five dimensions of health (mobility, self-care, usual activities, pain/discomfort, and anxiety/depression). For each dimension, participants are asked to select one of three responses to indicate the severity of problems experienced ‘today’ (e.g. no, moderate, or extreme pain or discomfort’) [16]. The EQ-5D-3L and more recent 5L version [17] are widely used in NZ and around the world in population-based health studies [18, 19], clinical settings, and policy making [20, 21]. Previously, amongst the Māori POIS cohort, we found the prevalence of EQ-5D-3L problems at 3-month post-injury to be substantially higher than pre-injury prevalence across each dimension (range: 24–70%), with increases ranging from threefold (anxiety/depression: 28%) to 11-fold (self-care: 24%) [11]. By 12-months post-injury, fewer Māori were experiencing problems than at 3-months post-injury but the prevalence for each outcome was still 2–4.5 times higher than pre-injury estimates (range: 7–55%) [12]. Given that the majority (75%) of the POIS 12-month respondents were not hospitalised for their injury, these findings highlight that injuries that might be considered ‘minor’ in terms of threat to life or hospitalisation can result in serious long-term functional limitations.

The aims of this paper are to (1) examine relationships between 12-month EQ-5D-3L outcomes and a range of pre-injury and injury-related factors and (2) identify pre-injury and injury-related predictors of problems with HRQoL at 12-months post-injury, for Māori POIS participants.

## Methods

Participants were recruited from five regions of NZ between December 2007 and August 2009. Individuals from these regions were eligible if they were aged between 18 and 64 years and on the Accident Compensation Corporation (ACC) entitlement claims register. ACC is NZ’s universal no-fault injury insurer and sole provider of injury insurance in NZ. Governed by a board accountable to the NZ Government, it is funded by a combination of compulsory levies (paid on income, fuel, and vehicle licencing fees) and government contributions. All NZ residents are covered by the scheme. Entitlement claimants are those who are eligible for loss of earnings and/or rehabilitation costs arising from their injury [23]. Claimants who were not citizens or residents of NZ and those whose injuries resulted from self-harm or sexual assault were ineligible.

Residents meeting the inclusion criteria who provided consent (obtained verbally) to take part were first interviewed, primarily by telephone, at approximately 3-months post-injury, then again at 12 and 24 months. More detailed information on the study protocols and participant characteristics are published elsewhere [7, 24]. Ethical approval was granted by the NZ Health and Disability Multi-region Ethics Committee (MEC/07/07/093).

The functional outcomes of interest in the present analyses were the five dimensions of the EQ-5D-3L [16] and a dichotomous measure, derived from summing participants’ responses to each dimension, scored as follows: no problems = 1, some problems = 2, and extreme problems = 3. Scores were then dichotomised into ≤ 6 (i.e. no problems or some problems on a single dimension) and ≥ 7 (extreme problems on at least one dimension or some problems on at least two dimensions).

Bivariate analyses were undertaken to examine the associations between each of the six binary outcomes and a range of independent variables. These variables were grouped into three categories: pre-injury sociodemographic, pre-injury health, and injury-related characteristics. Pre-injury sociodemographic variables included gender, age, occupation, and living arrangements, each obtained using the NZ Census 2006 questions [25], and adequacy of household income (Adequate: just enough, enough, more than enough; Not adequate: not enough), measured using an item from the NZ Household Economic Survey 2006–07 [26].

Ten pre-injury health-related variables were examined. Body Mass Index (BMI) was derived from participants’ self-reported height and weight. Where one or both of these were not reported, participants’ BMI was categorised as ‘unknown’. Prior chronic conditions were assessed using an instrument adapted from the NZ Health Survey 2006/07 [27]. Experience of a pre-injury depressive-type episode was determined using the DSM-III screening questions, with those answering ‘yes’ to any of the three questions classified as having had a depressive-type episode in the year prior to their injury [28]. General self-efficacy was assessed using a slightly modified version of the General Self-Efficacy Scale [29], with those scoring ≥ 26 considered to have good self-efficacy. Pre-injury HRQoL was measured using the EQ-5D-3L [16]. Hazardous drinking in the year prior to injury was assessed using the Alcohol Use Disorders Identification Test-Consumption (AUDIT-C) [30] and participants were also asked about the frequency of other recreational drug use during these 12 months. An item from the NZ Physical Activity Questionnaire was used to determine the daily frequency with which participants undertook moderate to vigorous physical activity in the week prior to their injury [31]. Participants were also asked about their satisfaction with social relationships pre-injury and if they were affected by a prior injury at the time of the injury for which they were recruited to POIS (POIS injury).

Injury-related characteristics included whether or not an individual’s POIS injury was due to physical assault, resulted in hospitalisation [32], was perceived (by the participant) to be a threat to life and/or of long-term disability, and/or was work-related. The association between injury severity, measured by the New Injury Severity Score (NISS) [33], and HRQoL was examined, as was HRQoL and participants’ trouble accessing healthcare for their injury, ascertained using the question: “Did you have trouble getting to or contacting health services?”.

Results of the bivariate analyses were used to construct separate multivariable models to assess associations with each of the six outcomes. Independent variables were selected for inclusion in the specific multivariable model if the respective association with the outcome of interest had a *p* value < 0.2. Potential predictor variables were then entered into a stepwise backward elimination regression analysis. Based on previous POIS analyses it was decided to force gender, age, injury severity, the corresponding pre-injury EQ-5D-3L dimension (e.g. pre-injury mobility for the mobility dimension outcome), and the number of days from injury to the 12-month interview into each model. Other variables were held in the final model if their associated *p* value < 0.15. Modified Poisson regression was used to estimate the relative risk (which is more intuitive and easier to interpret than odds ratios produced by logistic regression) and confidence interval for each retained variable using robust error variances [34]. Analyses were conducted using Stata/SE 13.1 [35].

## Results

The research team made contact with 4881 entitlement claimants and invited them to take part in the study. Of the 2856 participants completing the first (3-month post-injury) interview (a 59% participation rate), 566 (19.8%) reported Māori ethnicity, as per the NZ 2006 Census ethnicity question. The aim was to recruit 460 Māori, providing 80% power to detect differences in the prevalence of injury outcomes of at least 8% between Māori and non-Māori at the 5% level of significance. The 12-month interview was completed by 405 Māori participants. Four outcome variables had no missing data and two were missing a response from one participant only. No independent variable had more than 3.7% of participants’ data missing (median missingness across all independent variables: 0.5%).

### Identification of potential predictors of EQ-5D-3L outcomes at 12-months post-injury

The bivariate analyses showed that, at 12-months post-injury, females were at greater risk of experiencing problems across all five EQ-5D dimensions than males (Table 1). All but one of the associations were statistically significant, although gender and problems with self-care came close to reaching statistical significance (*p* = 0.06). The relationship between age and each EQ-5D-3L outcome was weaker. Whilst none of these associations were statistically significant, each had a *p* value < 0.2, supporting our inclusion of age in each of the multivariable models. The only other pre-injury sociodemographic variables to be included in multivariable models were adequacy of household income (usual activities, pain/discomfort, anxiety/depression) and occupational group (self-care).

**Table 1:** Prevalence of problems and relative risks of experiencing difficulties for EQ-5D-3L dimensions 12-months post-injury by pre-injury sociodemographic, pre-injury health and injury-related factors (unadjusted RR estimates)

*Characteristics*	*N*	EQ-5D-3L dimension														
		*Mobility*			*Self-care*			*Usual activities*			*Pain/Discomfort*			*Anxiety/Depression*		
Pre-injury sociodemographic		*N* = 404			*N* = 405			*N* = 405			*N* = 405			*N* = 405		
		*n *(%)	RR	*p*	*n *(%)	RR	*p*	*n *(%)	RR	*p*	*n *(%)	RR	*p*	*n *(%)	RR	*p*
*Gender*																
Male	254	64 (25)	ref	**0.04**	14 (6)	ref	**0.06**	67 (26)	ref	** < 0.01**	127 (50)	ref	**0.01**	42 (17)	ref	** < 0.01**
Female	151	52 (35)	1.4		16 (11)	1.9		68 (45)	1.7		95 (63)	1.3		44 (29)	1.8	
*Age (years)*																
18–29	102	19 (19)	ref	**0.05**	3 (3)	ref	**0.06**	25 (25)	ref	**0.06**	47 (46)	ref	**0.14**	15 (15)	ref	**0.09**
30–44	151	46 (30)	1.6		10 (7)	2.3		50 (33)	1.4		85 (56)	1.2		31 (21)	1.4	
45–64	152	51 (34)	1.8		17 (11)	3.8		60 (39)	1.6		90 (59)	1.3		40 (26)	1.8	
*Living situation*																
Alone	31	4 (13)	ref	0.21	3 (10)	ref	0.23	9 (29)	ref	0.85	17 (55)	ref	0.7	6 (19)	ref	0.74
Non-family	26	8 (31)	2.4		4 (15)	1.6		8 (31)	1.1		12 (46)	0.8		7 (27)	1.4	
Family	345	102 (30)	2.3		23 (7)	0.7		116 (34)	1.2		191 (55)	1.0		73 (21)	1.1	
*Adequacy of household income*																
Not adequate	46	15 (33)	ref	0.51	5 (11)	ref	0.34	19 (41)	ref	**0.19**	35 (76)	ref	** < 0.01**	14 (30)	ref	**0.09**
Adequate	358	100 (28)	0.9		25 (7)	0.6		115 (32)	0.8		186 (52)	0.7		72 (20)	0.7	
*Occupation*																
Professional	94	28 (30)	ref	0.78	13 (14)	ref	**0.1**	31 (33)	ref	0.91	50 (53)	ref	1.0	22 (23)	ref	0.82
Technical	93	25 (27)	0.9		5 (5)	0.4		30 (32)	1.0		50 (54)	1.0		18 (19)	0.8	
Trade/manual	172	50 (29)	1.0		10 (6)	0.4		58 (34)	1.0		91 (53)	1.0		33 (19)	0.8	
Unclassified	13	2 (15)	0.5		1 (8)	0.6		3 (23)	0.7		7 (54)	1.0		2 (15)	0.7	

Pre-injury health	*N*	*n *(%)	RR	*p*	*n *(%)	RR	*p*	*n *(%)	RR	*p*	*n *(%)	RR	*p*	*n *(%)	RR	*p*
*Body Mass Index*																
< 30	226	54 (24)	ref	**0.05**	8 (4)	ref	** < 0.01**	71 (31)	ref	0.28	121 (54)	ref	0.84	44 (19)	ref	**0.04**
30 +	160	55 (34)	1.4		18 (11)	3.2		55 (34)	1.1		90 (56)	1.1		34 (21)	1.1	
Unknown	19	7 (39)	1.6		4 (21)	5.9		9 (47)	1.5		11 (58)	1.1		8 (42)	2.2	
*Chronic conditions*																
None	197	53 (27)	ref	**0.17**	13 (7)	ref	**0.07**	53 (27)	ref	**0.01**	99 (50)	ref	** < 0.01**	33 (17)	ref	** < 0.01**
One	107	29 (27)	1.0		5 (5)	0.7		40 (37)	1.4		57 (53)	1.1		20 (19)	1.1	
Two or more	92	34 (37)	1.4		12 (13)	2.0		42 (46)	1.7		65 (71)	1.4		33 (36)	2.1	
*Depressive episode*																
No	292	73 (25)	ref	**0.01**	20 (7)	ref	0.48	90 (31)	ref	**0.1**	147 (50)	ref	** < 0.01**	49 (17)	ref	** < 0.01**
Yes	112	42 (38)	1.5		10 (9)	1.3		44 (39)	1.3		74 (66)	1.3		37 (33)	2	
*General self-efficacy*																
Poor	30	7 (23)	ref	0.5	2 (7)	ref	0.86	8 (27)	ref	0.43	15 (50)	ref	0.59	9 (30)	ref	0.21
Good	372	109 (29)	1.3		28 (8)	1.1		127 (34)	1.3		206 (55)	1.1		77 (21)	0.7	
*Hazardous drinker*																
No	118	40 (34)	ref	**0.14**	13 (11)	ref	**0.08**	47 (40)	ref	**0.08**	65 (55)	ref	1.0	28 (24)	ref	0.45
Yes	285	76 (27)	0.8		17 (6)	0.5		88 (31)	0.8		157 (55)	1.0		58 (20)	0.9	
*Relevant EQ-5D-3L dimension*																
No problems	[12]^a^	100 (26)	ref	** < 0.01**	27 (7)	ref	** < 0.01**	124 (32)	ref	**0.05**	192 (53)	ref	** < 0.01**	75 (20)	ref	** < 0.01**
Problems		16 (62)	2.3		3 (38)	5.5		11 (50)	1.5		29 (73)	1.4		11 (46)	2.3	
*Physical activity*																
< 5 days a week	172	50 (29)	ref	0.89	15 (9)	ref	0.44	55 (32)	ref	0.64	95 (55)	ref	0.84	37 (22)	ref	0.8
5–7 days a week	225	64 (28)	1.0		15 (7)	0.8		77 (34)	1.1		122 (54)	1.0		46 (20)	1.0	
*Prior injury*																
No	309	79 (26)	ref	**0.01**	23 (7)	ref	1.0	98 (32)	ref	**0.16**	155 (50)	ref	** < 0.01**	60 (19)	ref	**0.13**
Yes	94	37 (39)	1.5		7 (7)	1.0		37 (39)	1.2		67 (71)	1.4		25 (27)	1.4	
*Recreational drug use*																
No	297	90 (30)	ref	0.22	24 (8)	ref	0.4	99 (33)	ref	1.0	160 (54)	ref	0.52	58 (20)	ref	**0.16**
Yes	108	26 (24)	0.8		6 (6)	0.7		36 (33)	1.0		62 (57)	1.1		28 (26)	1.3	
*Satisfaction with social relationships*																
Not satisfied	25	8 (32)	ref	0.71	4 (16)	ref	**0.09**	8 (32)	ref	0.88	12 (48)	ref	0.5	7 (28)	ref	0.38
Satisfied	379	108 (29)	0.9		26 (7)	0.4		127 (34)	1.0		210 (55)	1.2		79 (21)	0.7	

Injury-related characteristics	*N*	*n *(%)	RR	*p*	*n *(%)	RR	*p*	*n *(%)	RR	*p*	*n *(%)	RR	*p*	*n *(%)	RR	*p*
*Assault*																
No	384	112 (29)	ref	0.47	28 (7)	ref	0.6	130 (34)	ref	0.52	213 (55)	ref	0.52	80 (21)	ref	0.24
Yes/maybe	19	4 (21)	0.7		2 (11)	1.4		5 (26)	0.8		9 (47)	0.9		6 (32)	1.5	
*Hospitalised*																
No	302	79 (26)	ref	**0.05**	26 (9)	ref	**0.13**	96 (32)	ref	0.25	166 (55)	ref	0.92	63 (21)	ref	0.75
Yes	103	37 (36)	1.4		4 (4)	0.5		39 (38)	1.2		56 (54)	1.0		23 (22)	1.1	
*Injury severity*																
1–3	176	49 (28)	ref	0.47	16 (9)	ref	0.59	63 (36)	ref	** < 0.01**	98 (56)	ref	**0.08**	42 (24)	ref	0.56
4–6	177	49 (28)	1.0		11 (6)	0.7		47 (27)	0.7		91 (51)	0.9		35 (20)	0.8	
> 6	44	16 (36)	1.3		3 (7)	0.7		23 (52)	1.5		30 (68)	1.2		8 (18)	0.8	
*Threat of disability*																
No	228	54 (24)	ref	**0.01**	12 (5)	ref	**0.06**	66 (29)	ref	**0.03**	111 (49)	ref	** < 0.01**	42 (18)	ref	**0.12**
Yes	173	62 (36)	1.5		18 (10)	2.0		68 (39)	1.4		110 (64)	1.3		43 (25)	1.3	
*Threat to life*																
No	345	93 (27)	ref	**0.06**	23 (7)	ref	0.24	111 (32)	ref	0.47	184 (53)	ref	**0.15**	71 (21)	ref	0.78
Yes	54	21 (39)	1.4		6 (11)	1.7		20 (37)	1.2		34 (63)	1.2		12 (22)	1.1	
*Accessing health services*																
No trouble	369	102 (28)	ref	**0.1**	27 (7)	ref	0.79	115 (31)	ref	** < 0.01**	203 (55)	ref	0.69	71 (19)	ref	** < 0.01**
Trouble	35	14 (40)	1.4		3 (9)	1.2		19 (54)	1.7		18 (51)	0.9		14 (40)	2.1	
*Work injury*																
No	264	71 (27)	ref	0.31	16 (6)	ref	**0.15**	83 (31)	ref	0.31	144 (55)	ref	0.93	50 (19)	ref	**0.15**
Yes	140	44 (32)	1.2		14 (10)	1.2		51 (36)	1.2		77 (55)	1.0		35 (25)	1.3	

Bold indicates independent variables where the *p* value of the bivariate analysis was < 0.2 and therefore the independent variable was selected for inclusion in the multivariable model analysis

^a^See Maclennan et al. [12] for pre-injury numbers specific to each EQ-5D-3L dimension

For each EQ-5D-3L dimension, individuals who reported problems pre-injury were at greater risk of having problems with that same dimension 12-month post-injury (Table 1). Those with a BMI ≥ 30 were at greater risk of experiencing problems with self-care than those with a lower BMI and those with ≥ 2 chronic conditions prior to their injury were at greater risk of experiencing problems with usual activities, pain/discomfort, and anxiety/depression than those with no chronic conditions. Problems with mobility were more likely to be experienced by those who were already affected by another injury at the time of their POIS injury and by those who had a depressive-type episode in the year prior to their POIS injury. Evidence for an association between pre-injury chronic conditions and problems with mobility and self-care was weaker but the variable met the threshold for inclusion in the multivariable models for these outcomes. Hazardous drinking (mobility, self-care, usual activities) and satisfaction with social relationships (self-care) also met the threshold for inclusion in the respective multivariable models.

Participants who perceived their injury to be a threat of long-term disability were more likely to be experiencing problems with mobility, usual activity, and pain/discomfort at 12-months post-injury (Table 1). The variable met the threshold for inclusion in each of these multivariable models. Those whose injury was of high severity (i.e. NISS > 6) were at greater risk of experiencing problems with usual activities than those whose injury was of lower severity, as were those who had trouble accessing healthcare services for their injury compared to those who did not have trouble. Participants who had trouble accessing healthcare services for their injury were also more likely to be experiencing problems with anxiety/depression at 12-months post-injury. None of the other associations between injury-related characteristics and HRQoL problems were statistically significant, but potential predictors included in other multivariable models were being hospitalised due to injury (mobility and self-care), injury severity (pain/discomfort), perceiving the injury as a threat to life (mobility), trouble accessing healthcare services (mobility), and the injury being work-related (self-care and anxiety/depression).

Results of the bivariate analyses of the EQ-5D-3L-summed score reflected those observed for the individual EQ-5D-3L dimensions (Table 2). Variables selected as potential predictors in the multivariable model, in addition to those forced in, were adequacy of household income, pre-injury chronic conditions, pre-injury depressive-type episode, hazardous drinking, affected by prior injury, hospitalisation due to injury, threat of disability, threat to life, and trouble accessing healthcare services.

**Table 2:** Prevalence and relative risks of HRQoL difficulties at 12-months post-injury by sociodemographic, pre-injury health, and injury-related factors (EQ-5D-summed score; unadjusted RR estimates)

EQ-5D-summed score ≥ 7 (N = 404)														
Pre-injury sociodemographic characteristics					Pre-injury health characteristics					Injury-related characteristics				
	*n*	(%)	RR	*p*		*n*	(%)	RR	*p*		*n*	(%)	RR	*p*
*Gender*					*Body Mass Index*					*Assault*				
Male	84	(33)	Ref	** < 0.01**	< 30	89	(39)	Ref	**0.08**	No	153	(40)	Ref	0.50
Female	78	(52)	1.6		30 +	62	(39)	1.0		Yes/maybe	9	(47)	1.2	
					Unknown	11	(61)	1.6						
					*Chronic conditions*									
					None	67	(34)	ref	** < 0.01**	*Hospitalised*				
					One	42	(39)	1.1		No	114	(38)	Ref	**0.11**
*Age (years)*					Two or more	53	(58)	1.7		Yes	48	(47)	1.2	
18–29	30	(30)	Ref	**0.02**	*Depressive episode*									
30–44	59	(39)	1.3		No	103	(35)	Ref	** < 0.01**					
45–64	73	(48)	1.6		Yes	58	(52)	1.5		*Injury severity*				
					*General self-efficacy*					1–3	72	(41)	Ref	**0.11**
					Poor	9	(30)	Ref	0.27	4–6	64	(36)	0.9	
					Good	153	(41)	1.4		> 6	23	(52)	1.3	
Living situation					*Hazardous drinker*									
Alone	10	(32)	Ref	0.57	No	54	(46)	Ref	**0.14**	*Threat of disability*				
Non-family	9	(35)	1.1		Yes	108	(38)	0.8		No	78	(34)	Ref	**0.01**
Family	141	(41)	1.3		*EQ-5D summed score*					Yes	83	(48)	1.4	
					≤ 6	143	(38)	Ref	**0.02**					
					≥ 7	17	(57)	1.5						
					*Physical activity*					*Threat to life*				
*Adequacy of household income*					< 5 days a week	70	(41)	Ref	0.75	No	131	(38)	Ref	**0.07**
Not adequate	24	(52)	Ref	**0.05**	5–7 days a week	88	(39)	1.0		Yes	27	(50)	1.3	
Adequate	137	(38)	0.7		*Prior injury*									
					No	115	(37)	Ref	**0.02**	*Accessing health services*				
					Yes	47	(50)	1.3		No trouble	143	(39)	Ref	**0.11**
					*Recreational drug use*					Trouble	18	(51)	1.3	
*Occupation*					No	118	(40)	Ref	0.87					
Professional	35	(37)	Ref	0.92	Yes	44	(41)	1.0						
Technical	37	(40)	1.1		*Satisfaction with social relationships*					*Work injury*				
Trade/manual	68	(40)	1.1		Not satisfied	9	(36)	Ref	0.67	No	101	(38)	Ref	0.33
Unclassified	4	(31)	0.8		Satisfied	153	(40)	1.1		Yes	60	(43)	1.1	

Bold indicates independent variables where the *p* value of the bivariate analysis was < 0.2 and therefore the independent variable was selected for inclusion in the multivariable model analysis

### Predictors of EQ-5D-3L outcomes at 12-months post-injury

#### Mobility

Three variables were found to have a strong association with mobility problems at 12-months post-injury (Table 3). Those aged 45–64 years at the time of injury were estimated to be 1.6 times more likely to experience problems with mobility compared to those aged 18–29 years. The risk of mobility problems was also higher amongst those who perceived their injury to be a threat of long-term disability and amongst those already experiencing problems with mobility pre-injury.

**Table 3:** Multivariate analysis of factors associated with problems in EQ-5D-3L dimensions and a summed score of 7 or more

Factors	EQ-5D-3L dimensions					EQ-5D-3L-summed score
	Mobility	Self-care	Usual activities	Pain/discomfort	Anxiety/depression	≥ 7
	*N* = 375	*N* = 360	*N* = 384	*N* = 382	*N* = 383	*N* = 381
	RR (95% CI)	RR (95% CI)	RR (95% CI)	RR (95% CI)	RR (95% CI)	RR (95% CI)
*Gender*^a^						
Male	1.00 *ref*	1.00 *ref*	1.00 *ref*	1.00 *ref*	1.00 *ref*	1.00 *ref*
Female	1.32 (0.96, 1.80)	1.60 (0.74, 3.46)	**1.68 (1.28, 2.19)**	**1.27 (1.07, 1.50)**	**1.90 (1.29, 2.79)**	**1.58 (1.25, 2.00)**
*Age*^a^ (years)						
18–29	1.00 *ref*	1.00 *ref*	1.00 *ref*	1.00 *ref*	1.00 *ref*	1.00 *ref*
30–44	1.50 (0.93, 2.42)	1.57 (0.47, 5.25)	1.36 (0.91, 2.03)	1.12 (0.87, 1.44)	1.46 (0.83, 2.56)	1.26 (0.87, 1.82)
45–64	**1.64 (1.03, 2.63)**	2.28 (0.72, 7.22)	1.34 (0.90, 2.01)	1.14 (0.88, 1.47)	1.70 (0.97, 2.97)	1.37 (0.96, 1.96)
*Pre-injury EQ-5D-3L score*^a^						
No problems/ ≤ 6	1.00 *ref*	1.00 *ref*	1.00 *ref*	1.00 *ref*	1.00 *ref*	1.00 *ref*
Problems/ ≥ 7	**1.85 (1.22, 2.63)**	**4.97 (2.26, 11.0)**	1.14 (0.73, 1.78)	1.02 (0.80, 1.30)	**1.79 (1.12, 2.85)**	1.02 (0.70, 1.48)
*Injury severity*^a^						
1–3	1.00 *ref*	1.00 *ref*	1.00 *ref*	1.00 *ref*	1.00 *ref*	1.00 *ref*
4–6	1.02 (0.72, 1.43)	0.82 (0.39, 1.72)	0.78 (0.57, 1.06)	0.99 (0.82, 1.19)	0.81 (0.55, 1.20)	0.93 (0.72, 1.21)
> 6	1.13 (0.72, 1.79)	0.87 (0.27, 2.83)	1.32 (0.93, 1.87)	1.22 (0.96, 1.55)	0.73 (0.36, 1.47)	1.27 (0.92, 1.74)
*Threat of disability*						
No	1.00 *ref*	1.00 *ref*	1.00 *ref*	1.00 *ref*	1.00 *ref*	1.00 *ref*
Yes	**1.40 (1.01, 1.94)**	1.94 (0.91, 4.13)	**1.31 (1.00, 1.71)**	**1.28 (1.08, 1.52)**	1.31 (0.91, 1.89)	**1.42 (1.12, 1.80)**
*Chronic conditions*						
None	(Dropped)	(Dropped)	1.00 *ref*	1.00 *ref*	1.00 *ref*	1.00 *ref*
One			1.27 (0.90, 1.79)	0.97 (0.77, 1.22)	1.01 (0.59, 1.72)	1.07 (0.78, 1.45)
Two or more			**1.54 (1.11, 2.14)**	1.17 (0.96, 1.44)	**1.75 (1.15, 2.67)**	**1.40 (1.06, 1.84)**
*Accessing health services*						
No trouble	(Dropped)	(Not entered)	1.00 *ref*	(Not entered)	1.00 *ref*	(Dropped)
Trouble			**1.48 (1.05, 2.09)**		**1.93 (1.23, 3.04)**	
*Body Mass Index*						
< 30	(Dropped)	1.00 *ref*	(Not entered)	(Not entered)	(Dropped)	(Dropped)
30 +		**2.80 (1.18, 6.62)**				
Unknown		**4.27 (1.40, 13.0)**				
*Depressive episode*						
No	1.00 *ref*	(Not entered)	(Dropped)	1.00 *ref*	(Dropped)	1.00 *ref*
Yes	1.31 (0.94, 1.82)			1.19 (0.99, 1.43)		**1.34 (1.04, 1.71)**
*Hospitalised*						
No	1.00 *ref*	(Dropped)	(Not entered)	(Not entered)	(Not entered)	(Dropped)
Yes	1.36 (0.98, 1.90)					
*Adequacy of household income*						
Not adequate	(Not entered)	(Not entered)	(Dropped)	1.00 *ref*	(Dropped)	(Dropped)
Adequate				**0.79 (0.65, 0.97)**		
*Prior injury*						
No	(Dropped)	(Dropped)	(Dropped)	1.00 *ref*	(Dropped)	(Dropped)
Yes				**1.29 (1.08, 1.56)**		
*Satisfaction with social relationships*						
Not satisfied	(Not entered)	1.00 *ref*	(Not entered)	(Not entered)	(Not entered)	(Not entered)
Satisfied		**0.28 (0.10, 0.81)**				
*Occupation*						
Professional	(Not entered)	1.00 *ref*	(Not entered)	(Not entered)	(Not entered)	(Not entered)
Technical		**0.34 (0.13, 0.86)**				
Trade/manual		**0.34 (0.15, 0.80)**				
Unclassified		0.72 (0.18, 2.89)				
*Recreational drug use*						
No	(Not entered)	(Not entered)	(Not entered)	(Not entered)	1.00 *ref*	(Not entered)
Yes					**1.76 (1.17, 2.64)**	

Bold indicates independent variables where the *p* value of the bivariate analysis was < 0.2 and therefore the independent variable was selected for inclusion in the multivariable model analysis

“Dropped” indicates a variable was dropped from a model during the stepwise backward elimination regression analysis, “Not entered” indicates that a variable was not entered into the model subjected to stepwise backward elimination regression analysis. Variables entered but that were not retained in any of the six models are not listed in the table

^a^Variable forced into final model

#### Self-care

Those experiencing problems with self-care prior to injury were more likely to be having problems with self-care at 12 months compared to those who were not (Table 3). Also at greater risk of self-care problems at 12 months were those with a BMI ≥ 30. Participants with technical or trade/manual jobs prior to injury were less likely to be having problems than those in professional occupations, as were those satisfied with their social relationships pre-injury compared to those who were not.

#### Usual activities

Females were at greater risk than males of experiencing problems undertaking usual activities at 12-months post-injury (Table 3). Those who had trouble accessing healthcare services for their injury or who perceived their injury to be a threat of long-term disability were also more likely to be experiencing problems, as were those with ≥ 2 chronic conditions compared to those with none. A comparison of the NISS “high”(> 6) and “moderate” (4–6) groups found that participants with a high severity injury were more likely than those who sustained an injury of moderate severity to be having problems with usual activities at 12 months (RR: 1.7; 95% CI 1.2, 2.5; *p* < 0.01); however, the greater risk for those with a high severity injury than those with a low severity injury (1–3) was not statistically significant (Table 3). Problems undertaking usual activities pre-injury did not predict problems at 12-months post-injury.

#### Pain/discomfort

At higher risk of pain/discomfort 12-months post-injury were females, those who perceived their injury to be a threat of long-term disability, and those already affected by an injury when sustaining their POIS injury (Table 3). Having adequate household income pre-injury was associated with a lower risk of experiencing pain/discomfort at 12-months post-injury.

#### Anxiety/depression

Females were more likely than males to experience anxiety/depression at 12-months post-injury, as were those with ≥ 2 chronic conditions prior to their injury than those with only one (RR: 1.7; 95% CI 1.1, 2.9) and those with none (Table 3). Participants who experienced anxiety/depression prior to their injury and those who had trouble accessing healthcare services for their injury were at greater risk of anxiety/depression 1-year post-injury. Recreational drug use in the year prior to injury also predicted anxiety/depression at 12 months.

#### EQ-5D-3L-summed score

Females were more likely than males to have a summed score of ≥ 7 at 12-months post-injury (Table 3). Those with ≥ 2 chronic conditions were at greater risk of a ≥ 7 summed score than those with none. Participants that had a pre-injury depressive-type episode and those who perceived their injury to be a threat of long-term disability were also more likely to have a summed score of ≥ 7.

## Discussion

This paper has identified factors associated with HRQoL problems at 12-months post-injury. It is the only study we are aware of that has specifically examined the range of health and well-being EQ-5D-3L outcomes for up to a year following injury for an Indigenous population. Furthermore, participants were a ‘general injury’ cohort that had sustained a broad range of injuries, in terms of type, body site, and severity, which did not result in hospitalisation for most. This and the sociodemographic and geographical characteristics of the regions participants were recruited from [7] mean the results are likely to apply to all populations of injured Māori aged 18–64 years from throughout New Zealand.

Whilst the recruitment of a sizeable cohort of injured Māori is a strength of our study, statistical precision, the degree of disaggregation within categorical variables, and the number of explanatory variables able to be included in multivariable models have been restricted by relatively limited statistical power. Therefore, some additional factors that might predict EQ-5D-3L outcomes 1-year post-injury may not have emerged from our analyses. The weaknesses of stepwise regression [e.g. 36] may also have resulted in our final models excluding some important predictors of 12-month problems and less important or nuisance variables being identified as predictors. We have attempted to minimise these limitations by ensuring our final models are not based purely on stepwise regression, complementing the approach with our content knowledge on the subject (e.g. initial variable selection for analysis and use of forced-in variables in regression models). The EQ-5D-3L-summed score outcome is also potentially limited in its utility—it gives equal weighting to the impact of each dimension on HRQoL and this may be an unreasonable assumption.

Another limitation of our study is the potential for attrition to have affected our observations. Over one quarter of the Māori cohort (28%) was lost to follow-up by the 12-month interview. However, analyses of the total POIS cohort (of which Māori participants comprise 20%) found no association between EQ-5D-3L outcomes at 3 months and non-participation at 12 months [37]. This suggests that attrition is unlikely to be impacting on our estimates. Measurement and response errors could also be influencing our findings. Pre-injury health information was collected retrospectively at the first interview (3-months post-injury). Participants may have provided more positive pre-injury health status information not cognisant that they were comparing it to their post-injury health state. However, previous POIS analyses suggest that any error from using recalled pre-injury health information is likely to be negligible [38]. Similarly, we believe any response error is likely to be minor. It was made clear to participants from the outset that the study was being conducted independent of ACC and no individual data would be shared with the organisation, meaning participants’ responses would have no impact on their compensation claims or healthcare provision as ACC would not be able to identify individual responses. Participant self-selection into the cohort may be impacting our results if they differed from those who did not take part with respect to our outcomes of interest.

Gender was one of the more common predictors of 12-month problems (usual activities, pain/discomfort, anxiety/depression, ≥ 7). There is no obvious explanation for why females are at greater risk of problems than males. Whilst males were more likely to have been lost to follow-up by the 12-month interview [37], the finding of no association between EQ-5D-3L outcomes at 3 months and non-participation at 12 months suggests that the finding is unlikely to be due to attrition. Perhaps, for some dimensions, it is due to role differences. It is possible that women are more likely than men to do activities that are hampered by injury or they may undertake activities considered “usual” more often than men do and are therefore more likely to report problems doing these.

Other common predictors of 12-month problems included pre-injury HRQoL. Those experiencing problems with mobility, self-care, or anxiety/depression prior to their injury were at greater risk of experiencing problems with the respective outcome at 12 months. Perceived threat of long-term disability was associated with a higher risk of problems with four of the six EQ-5D-3L outcomes: mobility, usual activities, pain/discomfort, and the summed score of ≥ 7. As with gender, the exact mechanism underlying these relationships is unclear. Having two or more chronic conditions pre-injury was associated with a higher risk of problems with three outcomes (usual activities, anxiety/depression, ≥ 7). Chronic conditions have predicted other adverse outcomes in previous POIS analyses [13, 39] and we postulate that the burden of such conditions may impact on injury recovery.

Trouble accessing healthcare services for one’s injury predicted problems with usual activities and with anxiety/depression. This has important implications given the adverse impact that psychological morbidity has been found to have on injury recovery [40] and outcomes [41, 42]. Our study found that having a depressive-type episode in the year prior to injury increased the risk of having a summed EQ-5D score of ≥ 7 at 12-months post-injury. This further demonstrates the need for efforts to ensure improved and timely access to high quality and culturally appropriate health services for Māori in order to reduce health and well-being inequities. Previous research has found that Māori have lower rates of access to ACC and health services and are subject to lower quality of care and have less equitable outcomes when services are accessed than non-Māori [43–46]. One might expect that adequate household income would facilitate access to care but perhaps the care received does not optimise recovery for Māori. However, it may enable access to medicinal treatments that help relieve pain and discomfort, the only problem at 12-months post-injury for which adequate household income protected against for Māori POIS participants.

There is merit in investigating further the interplay between trouble accessing healthcare services and adequacy of household income and their impact on EQ-5D-3L outcomes post-injury. Qualitative interviews with Māori POIS participants revealed that access to health services went beyond the issue of cost and included a lack of suitable times outside of work hours for treatment and rehabilitation appointments and issues with transport availability and/or difficulties in arranging childcare to attend these (Wyeth et al., in preparation). POIS-10 Māori [22] is investigating further reasons people have difficulty accessing healthcare for their injury.

An unexpected finding in this study was a ‘J-shaped’ relationship between NISS and problems with usual activities at 12-months post-injury. Our previous analyses of Māori data show that the proportion of participants who sustained a low severity injury (NISS 1–3) was slightly lower amongst 12-month respondents [12] than 3-month respondents [11]. The prevalence of EQ-5D-3L problems at 3-months post-injury was lower amongst those sustaining a low severity injury [11]. If those who sustained a low severity injury and who had no functional problems at 3 months were more likely to be lost to follow-up at the 12-month interview, then this would have biased our prevalence estimate for functional problems upwards amongst the low severity injury sub-group, leading to our observed ‘J-shaped’ relationship. However, the finding of no relationship for the overall cohort between 3-month EQ-5D problems and non-participation at 12 months does not support this hypothesis. Furthermore, no association was observed between injury severity and non-participation at 12 months [37]. People’s definition of a usual activity may vary or a person’s definition may change depending on the severity of the injury they sustain. For example, those sustaining a moderate injury may no longer be able to do what they formerly considered a usual activity and therefore no longer consider it a usual activity. Those with a low severity injury may still be able to do such an activity and still consider it a usual activity, but may continue to have trouble undertaking it. On the other hand, the finding may be due to our limited statistical power.

Another interesting finding was the lower risk of problems with self-care at 12 months amongst those working in a technical or trade/manual occupation compared to those in other occupations. These individuals may receive greater support from ACC or their employer to rehabilitate for their injury, which may be vital to them returning to work. Being satisfied with one’s social relationships pre-injury also protected against problems with self-care at 12 months. Again, this may reflect having greater support to rehabilitate for injury, although, if this is the case, it begs the question, as it does for occupational group, why it was not protective against problems for other EQ-5D-3L outcomes. Few participants experienced problems with self-care at 12 months, so findings for this dimension need to be treated with caution. This includes our finding that having a pre-injury BMI ≥ 30 increased the risk of problems with self-care, although this same relationship was also found in analyses undertaken on the total POIS cohort [14].

Age predicted only the mobility outcome, with those aged 45–64 years at greater risk of problems than those aged 18–29 years. Recreational drug use and prior injury also predicted problems with a single EQ-5D-3L outcome only: anxiety/depression and pain/discomfort, respectively. Whilst we are uncertain why recreational drug use in the year prior to injury increased the risk of anxiety/depression 1 year after injury, our finding on prior injury is consistent with previous research showing subsequent injuries to have a more deleterious impact on work participation outcomes than an initial injury event [47–49] and more than for those only experiencing an initial injury [50]. Perhaps there is a cumulative effect of sustaining multiple injuries in a specific period in terms of suffering pain or discomfort.

The ability to compare our findings with other studies is restricted by a lack of similar “all injury general population” studies focusing specifically on Indigenous populations. This highlights the importance of researchers planning and designing studies and ensuring the resources and appropriate methods, to recruit sufficiently large numbers of Indigenous participants in studies examining population health issues. This is necessary to produce the robust findings vital to facilitating health equity for Indigenous populations.

Importantly, our findings are currently being used to inform the development of an intervention to help identify injured Māori who would benefit early on from greater care and support in order to optimise recovery and reduce the chance of adverse outcomes at 12-months post-injury. They will also inform future Māori EQ-5D-3L analyses and comparisons with 12-year post-injury outcomes for injured Māori as part of the POIS-10 Māori study [22] for which data collection has recently been completed (July 2021) and is now being prepared for analyses. Current and future findings will provide useful information for ACC and health providers as to how they can optimise care and improve outcomes for injured Māori.

## Data Availability

The study data cannot be shared due to ethical constraints.

## Code availability

Code for the statistical analyses is available from the corresponding author upon reasonable request.

## References

[1] Anderson I, Robson B, Connolly M, Al-Yaman F, Bjertness E, King A, Tynan M, Madden R, Bang A, Coimbra CEA, Pesantes MA, Amigo H, Andronov S, Armien B, Obando DA, Axelsson P, Bhatti ZS, Bhutta ZA, Bjerregaard P (2016). Indigenous and tribal peoples’ health (The Lancet-Lowitja Institute Global Collaboration): A population study. The Lancet.

[2] Health Quality and Safety Commission New Zealand (2019). *He matapihi ki te kounga o ngā manaakitanga ā-hauora o Aotearoa 2019: A window on the quality of Aotearoa New Zealand's health care 2019*. Health Quality and Safety Commission New Zealand, Wellington

[3] Steyn N, Binny RN, Hannah K, Hendy SC, James A, Kukutai T, Lustig A, McLeod M, Plank MJ, Ridings K, Sporle A (2020). Estimated inequities in COVID-19 infection fatality rates by ethnicity for Aotearoa New Zealand. New Zealand Medical Journal.

[4] Yashadhana A, Pollard-Wharton N, Zwi AB, Biles B (2020). Indigenous Australians at increased risk of COVID-19 due to existing health and socioeconomic inequities. The Lancet Regional Health—Western Pacific.

[5] World Health Organisation (2021). It’s time to build a fairer, healthier world for everyone, everywhere World Health Day 2021.

[6] World Health Organization (1978). *Primary health care: report of the International Conference on Primary Health Care, Alma Ata, USSR, 6–12 September 1978*. World Health Organization, Geneva

[7] Derrett S, Langley J, Hokowhitu B, Ameratunga S, Hansen P, Davie G, Wyeth E, Lilley R (2009). Prospective outcomes of injury study. Injury Prevention.

[8] Wyeth EH, Derrett S, Hokowhitu B, Hall C, Langley J (2010). Rangatiratanga and Oritetanga: Responses to the Treaty of Waitangi in a New Zealand study. Ethnicity & Health.

[9] Wyeth EH, Derrett S, Hokowhitu B, Samaranayaka A (2013). Indigenous injury outcomes: Life satisfaction among injured Māori in New Zealand three months after injury. Health & Quality of Life Outcomes.

[10] Wyeth EH, Maclennan B, Lambert M, Davie G, Lilley R, Derrett S (2018). Predictors of work participation for Māori 3 months after injury. Archives of Environmental & Occupational Health.

[11] Maclennan B, Wyeth E, Hokowhitu B, Wilson S, Derrett S (2013). Injury severity and three-month outcomes among New Zealand Māori: Results from a New Zealand prospective cohort study. New Zealand Medical Journal.

[12] Maclennan B, Wyeth E, Davie G, Wilson S, Derrett S (2014). Twelve-month post-injury outcomes for Māori and non-Māori: Findings from a New Zealand cohort study. Australian and New Zealand Journal of Public Health.

[13] Wyeth EH, Samaranayaka A, Davie G, Derrett S (2017). Prevalence and predictors of disability for Māori 24 months after injury. Australian and New Zealand Journal of Public Health.

[14] Langley J, Davie G, Wilson S, Lilley R, Ameratunga S, Wyeth E, Derrett S (2013). Difficulties in functioning 1 year after injury: The role of preinjury sociodemographic and health characteristics, health care and injury-related factors. Archives of Physical Medicine and Rehabilitation.

[15] Rabin R, de Charro F (2001). EQ-5D: A measure of health status from the EuroQol Group. Annals of Medicine.

[16] EuroQol Group (2013). *EQ-5D-3L|About*. EuroQol Group. Retrieved August 30, 2021, from https://euroqol.org/eq-5d-instruments/eq-5d-3l-about/

[17] EuroQol Group (2021). *EQ-5D-5L|About*. EuroQol Group. Retrieved September 20, 2021, from https://euroqol.org/eq-5d-instruments/eq-5d-5l-about/

[18] Pequeno NPF, Cabral NLdA, Marchioni DM, Lima SCVC, Lyra CdO (2020). Quality of life assessment instruments for adults: A systematic review of population-based studies. Health and Quality of Life Outcomes.

[19] Geraerds AJLM, Richardson A, Haagsma J, Derrett S, Polinder S (2020). A systematic review of studies measuring health-related quality of life of general injury populations: Update 2010–2018. Health and Quality of Life Outcomes.

[20] Devlin NJ, Krabbe PFM (2013). The development of new research methods for the valuation of EQ-5D-5L. The European Journal of Health Economics.

[21] Devlin NJ, Brooks R (2017). EQ-5D and the EuroQol Group: Past, present and future. Applied Health Economics and Health Policy.

[22] Wyeth EH, Derrett S, Nelson V, Bourke J, Crengle S, Davie G, Harcombe H (2021). POIS-10 Māori: Outcomes and experiences in the decade following injury. Methods and Protocols.

[23] Accident Compensation Corporation (2019). Supporting the Kiwi way of life: Annual report 2019.

[24] Derrett S, Davie G, Ameratunga S, Wyeth E, Colhoun S, Wilson S, Samaranayaka A, Lilley R, Hokowhitu B, Hansen P, Langley J (2011). Prospective outcomes of injury study: Recruitment, and participant characteristics, health and disability status. Injury Prevention.

[25] Statistics New Zealand—Tatauranga Aotearoa (2006). *2006 Census of population and Dwellings*. Statistics New Zealand. Retrieved June 18, 2020, from http://archive.stats.govt.nz/Census/about-2006-census/2006-census-questionnaires.aspx#gsc.tab=0

[26] Statistics New Zealand—Tatauranga Aotearoa (2007). *Household Economic Survey 2006–07*. Statistics New Zealand. Retrieved June 25, 2013, from http://www.stats.govt.nz/browse_for_stats/income-and-work/Income/HES-06-07-datasets/printable-questionnaires.aspx

[27] Ministry of Health—Manatū Hauora (2010). 2006/07 New Zealand health survey: Adult questionnaire. Ministry of Health. Retrieved June 25, 2013, from http://www.health.govt.nz/nz-health-statistics/national-collections-and-surveys/surveys/technical-documentation

[28] American Psychiatric Association Committee of Nomenclature and Statistics (1980). Diagnostic and statistical manual of mental disorder.

[29] Schwarzer R, Jerusalem M, Weinman J, Wright S, Johnston M (1995). Generalized self-efficacy scale. Measures in health psychology: A user’s portfolio casual and control beliefs.

[30] Bush K, Kivlahan DR, McDonell MB, Fihn SD, Bradley KA (1998). The AUDIT alcohol consumption questions (AUDIT-C): An effective brief screening test for problem drinking. Archives of Internal Medicine.

[31] Sport and Recreation New Zealand (2004). The New Zealand Physical Activity Questionnaires. SPARC, Wellington, New Zealand

[32] Ministry of Health—Manatū Hauora (2012). *National Minimum Dataset (Hospital Events) data dictionary*. Ministry of Health. Retrieved June 25, 2013, from http://www.health.govt.nz/publication/national-minimum-dataset-hospital-events-data-dictionary

[33] Stevenson M, Segui-Gomez M, Lescohier I, Di Scala C, McDonald-Smith G (2001). An overview of the injury severity score and the new injury severity score. Injury Prevention.

[34] Zou G (2004). A modified poisson regression approach to prospective studies with binary data. American Journal of Epidemiology.

[35] StataCorp, L. P. (1985–2013). 13.1 ed. College Station, TX, USA

[36] Smith G (2018). Step away from stepwise. Journal of Big Data.

[37] Langley JD, Lilley R, Wilson S, Derrett S, Samaranayaka A, Davie G, Ameratunga SN, Wyeth EH, Hansen P, Hokowhitu B (2013). Factors associated with non-participation in one or two follow-up phases in a cohort study of injured adults. Injury Prevention.

[38] Wilson R, Derrett S, Hansen P, Langley J (2012). Retrospective evaluation versus population norms for the measurement of baseline health status. Health & Quality of Life Outcomes.

[39] Harcombe H, Davie G, Wyeth E, Ameratunga S, Powell D, Derrett S (2020). Predictors of severe or multiple subsequent injuries over 24 months among an already-injured cohort in New Zealand. Injury.

[40] Kellezi B, Coupland C, Morriss R, Beckett K, Joseph S, Barnes J, Christie N, Sleney J, Kendrick D (2017). The impact of psychological factors on recovery from injury: A multicentre cohort study. Social Psychiatry and Psychiatric Epidemiology.

[41] Derrett S, Wilson S, Samaranayaka A, Langley J, Wyeth E, Ameratunga S, Lilley R, Davie G, Mauiliu M (2013). Prevalence and predictors of disability 24-months after injury for hospitalised and non-hospitalised participants: Results from a longitudinal cohort study in New Zealand. PLoS ONE.

[42] Harcombe H, Davie G, Wyeth E, Samaranayaka A, Derrett S (2018). Injury upon injury: A prospective cohort study examining subsequent injury claims in the 24 months following a substantial injury. Injury Prevention.

[43] Mauri Ora Associates (2010). *Māori Experience of ACC: Mauri Ora Associates Final Report for the Department of Labour*. Mauri Ora Associates

[44] Rumball-Smith JM (2009). Not in my hospital? Ethnic disparities in quality of hospital care in New Zealand: A narrative review of the evidence. The New Zealand Medical Journal.

[45] Ministry of Health—Manatū Hauora (2014). Annual update of key results 2013/14: New Zealand health survey.

[46] Bradley, A. (2021). *ACC biased against women, Māori and Pasifika—agency's own analysis shows*. Radio New Zealand. Retrieved September 1, 2021, from https://www.rnz.co.nz/news/national/445178/acc-biased-against-women-maori-and-pasifika-agency-s-own-analysis-shows

[47] Wasiak R, Kim J, Pransky G (2006). Work disability and costs caused by recurrence of low back pain: Longer and more costly than in first episodes. Spine.

[48] Ruseckaite R, Collie A (2011). Repeat workers’ compensation claims: Risk factors, costs and work disability. BMC Public Health.

[49] Ruseckaite R, Collie A (2013). The incidence and impact of recurrent workplace injury and disease: A cohort study of WorkSafe Victoria, Australia compensation claims. British Medical Journal Open.

[50] Wilson S, Davie G, Harcombe H, Wyeth EH, Cameron I, Derrett S (2018). Impact of further injury on participation in work and activities among those previously injured: Results from a New Zealand prospective cohort study. Quality of Life Research.

